# Optimal Inter-Organization Control of Collaborative Advertising with Myopic and Far-Sighted Behaviors

**DOI:** 10.3390/e23091144

**Published:** 2021-08-31

**Authors:** Yinfei Xu, Yafei Zu, Hui Zhang

**Affiliations:** 1 School of Information Science and Engineering, Southeast University, Nanjing 210096, China; yinfeixu@seu.edu.cn; 2 School of Economics and Management, Nanjing University of Science and Technology, Nanjing 210094, China; 3 Inspur Academy of Science and Technology, Jinan 250000, China

**Keywords:** collaborative advertising, differential game, inter-organization control, myopic/far-sighted behaviors

## Abstract

This paper investigates the optimal inter-organization control of collaborative advertising considering the myopic and far-sighted behaviors. Taking a two-echelon supply chain as an example, four kinds of differential game models including myopic Stackelberg game, far-sighted Stackelberg game, myopic cooperative game and far-sighted cooperative game are studied. The results show that the optimal advertising efforts of both manufacturer and retailer in the myopic situation decrease with time. But they remain constant in the far-sighted situation. The Pareto improvement applies to both game players from the non-cooperative game to the cooperative game. The numerical analyses are conducted to further investigate the theoretical results and to guide the inter-organization control of collaborative advertising in practice.

## 1. Introduction

Faced with fierce competing environment, more and more firms tend to act myopically to search for the maximization of short-term profits. However, based on the value-added theory, firms, managers should focus more on the profit in the long run and make decisions far-sightedly. Prior studies explore the decision rules of myopic and far-sighted behaviors and indicate that the difference between these two behaviors is that, a myopic manager makes decision only considering the impact in a finite period, while a far-sighted manager will take the further influence of her decisions into account in an infinite period (Chakravarti et al. [1]; Hauser et al. [2]; Liu et al. [3]; Che et al. [4]). Most of these studies focus on the impact of myopic/far-sighted behaviors on the strategies such as pricing, quality, low-carbon production and so on. However, there are few studies consider their influence on the collaborative advertising activity. Advertising is one of the most important activities for a firm to sell products. It affects consumers’ buying behaviors, brand image, and firms’ profits. Advertising activities include the national advertising and the local advertising (Huang et al. [5]). In the national market, the manufacturer is responsible to cultivate the product’s impression in consumers. And in the local market, the retailer aims to let consumers to buy the products right away. Nowadays, with the rapid expansion of supply chain all over the world, the collaborative advertising activity between the manufacturer and the retailer (both the national and local advertising) becomes more crucial and common in practice. Hence studying the impact of myopic/far-sighted behaviors on the collaborative advertising activities in the supply chain has critical meanings in both practice and theory.

Price is the core factor that affects the market demand significantly. The classical economics theory indicates that a lower price results in a higher market demand. However, when we consider the effect of reference price, things may be different. Reference price is a kind of price concept existing in consumers’ minds (Fibich et al. [6]). It mainly depends on the past price of product and can affect consumers’ choices. Specifically, when the real price is higher than its reference price in consumers’ minds, consumers may generate the feeling of loss (Lattin and Buckin [7]; Nasiry and Popescu [8]). Under this situation, they may buy less products and the demand will decrease. Hence the impact of price on the demand need to consider the reference price effect at the same time. However, most prior studies on the pricing strategy mainly focus on the static decisions (Feng et al. [9]). While with the fast-changing business environment, the dynamic pricing strategy is more beneficial for firms to maximize their profits. Moreover, as we mentioned above, the collaborative advertising also has important impact on the market demand. Studies on the collaborative advertising and reference price effect mainly consider their individual effects regardless of the synthesized impacts on the supply chain. Hence taking the synthesized effect of collaborative advertising and reference price into account is critical in the analysis of behavior preferences in the supply chain.

Based on the analysis above, using the differential game method, this paper studies the optimal inter-organization control of collaborative advertising with myopic and far-sighted behaviors in a supply chain. Specifically, it analyzes how the manufacturer can motivate the retailer to make more efforts on local advertising and further enhance the product goodwill and the total profit of supply chain. This paper is organized as follows. Section 2 reviews the relevant literature in four research fields. And the model framework is presented in Section 3. In Section 4 and Section 5, four kinds of differential game models are developed based on the myopic/far-sighted behaviors in the supply chain and the game structures. The numerical analysis is conducted in Section 6 and Section 7 finally presents the conclusions.

## 2. Literature Review

This paper is related to four fields of research including the advertising coordination, reference price, myopic/far-sighted behaviors and the application of game theory.

Advertising coordination is critical in supply chain management. Many scholars find the significant relationship between advertising coordination and profit (Dorfman and Steiner [10]; Chioveanu [11]). Based on the prior studies, He et al. [12] further explore the influence of dynamic advertising on pricing decisions in the supply chain. In the aspect of game structure, Xie and Wei [13] study the equilibriums of advertising strategies in the situation of non-cooperation game and cooperation game. Chaab and Rasti-Barzoki [14] further compare the advertising strategies between the Stackelberg game and cooperative game. From the perspective of supply chain structure, Karray and Amin [15] find that the collaborative advertising may not have positive effect on the total profit when there are many competing retailers. In the aspect of advertising type, Zhang et al. [16] study the national and local advertising simultaneously. They indicate that when consumers are significant influenced by product’s reference price, it would be better for the supply chain to choose more national advertising. Those prior research mainly focuses on the effect of collaborative advertising on supply chain profit. However, the behavior preference of partner firms, especially the myopic/far-sighted behaviors, will also affect the advertising strategies and the total profit of supply chain. This field of research is still insufficient.

The second stream of literature related to our work is about the reference price. Popescu and Wu [17] develop a multi-period model to study the reference price effect. They indicate that firms should fully consider the long-term profit when they make decisions. Fibich et al. [18] find that when consumers’ loss effect is higher than gain effect, the equilibrium price will remain unchanged. This result is consistent to the study of Popescu and Wu [17]. Based on their model, Geng et al. [19] take the supply chain structure into account and extend the static model to a dynamic one. Zhang et al. [20] assume a bilateral monopoly market and develop a differential model to study the pricing strategies in the supply chain. They find that a higher initial reference price would be helpful to improve the supply chain’s total profit. Those prior studies mainly focus on the impact of reference price on supply chain’s pricing strategies. The research on the impact of reference price effect on other factors such as advertising, quality and so on are still under development. Especially, as the collaborative advertising is one of the most critical activities for the supply chain to improve profit, the integrated effect of reference price and advertising is becoming more and more meaningful and urgent.

The third stream of literature related to our work is about the behavior preference (especially the myopic/far-sighted behaviors) of partner firms in the supply chain. Taboubi and Zaccour [21] find that a myopic retailer tends to set a lower price and she will not work hard. Under this situation, the manufacturer needs to make more effort. Chiang [22] also indicate that it would be better for the firm to make decisions far-sightedly. This result is consistent to the findings of Taboubi and Zaccour [21]. However, Gutierrez and He [23] show that the manufacturer will gain from a myopic retailer. This result is inconsistent with Taboubi and Zaccour [21] and Chiang [22]. Benchekroun et al. [24] further compare the effect of myopic and far-sighted behaviors on firms’ optimal strategies. Their result show that, it would be beneficial for both partner firms to choose the myopic decisions when the reference price is low. Liu et al. [3] formulate a two-echelon supply chain model and find that far-sighted behaviors is less sensitive to the quality and more sensitive to the price. Most of these prior studies indicate that the myopic behavior will ignore the further influence of current decision. However, in practice, the myopic behavior is more like a matter of short finite time interval. Myopic firms will also consider the influence of current decision. However, the influencing period in their minds is relatively short.

The fourth stream of literature related to our work is about the game theory. Prior literatures mainly focus on the applications such as profit/cost allocation, contract coordination and so on (Nagarajan and Sosic [25]; Meca and Sosic [26]; Wang et al. [27]). In the supply chain, the common game structures include the Nash game, Stackelberg game and cooperative game. For example, He et al. [12] formulate a Stackelberg game model in a two-echelon supply chain. They find that it would be better for the whole supply chain when the manufacturer provides incentives to the retailer. In the aspect of differential game model, Martín-Herrán and Taboubi [28] use a it to study the dynamic pricing strategies and their influences on profits in the situations of cooperative and non-cooperative game. Specifically, Zhang et al. [16] use the differential game model to study the interaction of dynamic advertising and reference price, and their overall impacts on the profits of supply chain. They assume that the advertising efforts of partner firms can affect both reference price and product goodwill directly. However, in practice, consumers may not perceive the advertising effort of firms well. It results in that most of the advertising effort cannot affect product goodwill directly. Our paper develops a differential game model to study the myopic/farsighted behaviors in collaborative advertising with reference price. It improves the prior game models on the relationship between advertising effort and product goodwill.

In our previous work [29], the impact of dynamic advertising coordination on supply chain was studied from the perspective of different game structures (including Nash non-cooperative game, Stackelberg game and cooperative game). The game players are assumed to be rational and neutral in this study, namely they have no preference when making decisions. The impact of firm’s behavior preference on dynamic advertising was ignored. However, in practice, partner firms in supply chain have specific behavior preference most of the time. And such behaviors may affect firm’s decision and the final profit significantly. Hence in this paper, we fully consider the influence of firm’s behavior characteristics on their advertising strategies and study the situation when the partner firms are myopic and far-sighted. This new situation is closer to the reality and more meaningful than work [29]. Moreover, ref. [30] studied the impact of behavior choice on firm’s profit. It assumed the difference between myopic and far-sighted behavior was that whether the firms consider the further influence of their current decisions. Such definition can be expressed as the different constraint conditions in firms’ objective functions. However, in practice, the myopic firm is more likely to make decisions focusing on a short sales period. In this finite period, myopic firms will also consider their decisions’ influences. Namely, the difference between the myopic and far-sighted behavior is more likely to be their decision intervals, not the constraint conditions. Thus, in this paper, we develop a new model to define the myopic and far-sighted behavior in terms of decision intervals and further explore their impacts on firms’ optimal strategies and profits. This new definition depicts the characteristics of myopic and far-sighted behavior more clearly. Those two improvements above lead to that the solutions in this paper have more practical guiding significance.

## 3. Model Framework

**Figure 1:**
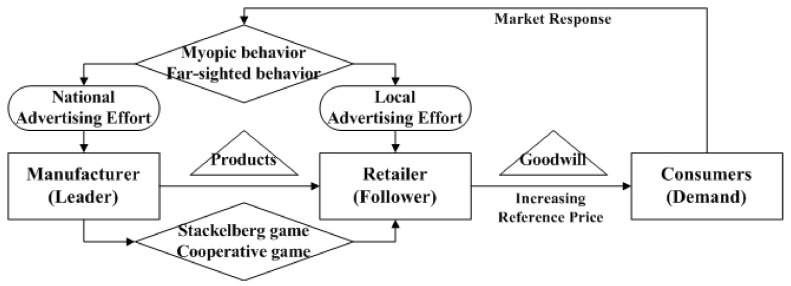
Mechanism of collaborative advertising with reference price effect.

Assume that there is a two-echelon supply chain consisting of one manufacturer and one retailer. The manufacturer owns the critical technology and acts as the leader in their relationship. The retailer is the follower. Consumers also play the significant role in this framework due to their purchasing decisions. Consumers’ decisions are affected by the goodwill and reference price in their minds. For convenience, problems of production cost, inventory, price and so on are omitted in this paper. The mechanism of this problem can be described as Figure 1. The notations and definitions in this differential game analysis used in this paper are shown in Table 1.

Although both manufacturer and retailer make efforts on advertising, their directions are not the same. The manufacturer is responsible for advertise nationwide to create the good image for products. The retailer is in charge of the local advertising which will result in the instantaneous promotion. When manufacturer and retailer make efforts on advertising, consumers will form the goodwill that the products they sold are excellent. However, when there are no efforts on advertising, products’ goodwill may decay due to consumers’ forgetfulness. All this process is varied with time. Hence products’ goodwill can be written as

(1) $$\dot{W}\left(t\right)=\theta_{M}E_{M}\left(t\right)+\theta_{R}E_{R}\left(t\right)-\delta W\left(t\right),\;W\left(0\right)=W_{0}\geq 0.$$

According to practice, a higher goodwill is beneficial to increase the reference price in consumers’ minds. Because consumers tend to think the product is of high quality. Furthermore, when the real price is lower than the reference price, consumers tend to perceive a sense of gain. Then they are likely to reduce the reference price in their minds. Hence the differential expression of reference price can be given as

(2) $$\dot{r}\left(t\right)=\gamma \left(p(t)-r(t)\right)+\sigma W\left(t\right),\;r\left(0\right)=r_{0}\geq 0.$$

In this study, we aim to solve the problem of collaborative advertising, the decision of retail price is not the key point. And one can calculate the specific market price in our model by partial derivatives. In the aspect of practice, it is unusual to change the market price with time so frequently. The classical study of promotion effect conducted by Lattin and Bucklin [7] had proved that a frequently changing price may lead to a bad image of the companies and a decreasing market demand on the contrary. In the competitive market, the market equilibrium price just can be influenced by one company slightly, so it is unwise to change the price regardless of the other market factors. Hence both manufacturer and retailer are not willing to change the market price frequently in theory and practice. As we have mentioned above, when the real price is lower than the reference price, consumers tend to perceive a sense of gain and purchase more products. Product’s goodwill is also positive to its market sales. Hence the market demand can be expressed as

(3) $$D\left(t\right)=D_{0}+\alpha \left(r(t)-p(t)\right)+\beta W\left(t\right).$$

The cost of both national advertising and local advertising are set in a quadratic form to meet the marginal diminishing effect. An upper bound *M* of the effort is assumed, so that $0\leq E_{M}\left(t\right),E_{R}\left(t\right)\leq M$. Hence the effort cost of manufacturer and retailer can be given as

(4) $$C_{M}=\frac{\mu_{M}}{2}E_{M}^{2}\left(t\right),\;C_{R}=\frac{\mu_{R}}{2}E_{R}^{2}\left(t\right).$$

## 4. Stackelberg Game Situations

In a Stackelberg game situation, there exists a status gap between the manufacturer and the retailer. With the core technology, the manufacturer plays the role of leader in the whole supply chain, while the retailer is the follower. We present the analyses of myopic and far-sighted scenarios, respectively, considering that the behavioral tendencies of the manufacturer and the retailer have an important influence on their optimal strategies. Both of them coordinate with each other to promote the market demand currently or in the long run. The game sequence is: firstly, the manufacturer makes a decision on his own effort on national advertising $E_{M}$, and the cost subsidy rate ϕ(t) to the retailer on his local advertising effort. Then, after observing the action of manufacturer, the retailer decides his own effort on local advertising $E_{R}$.

### 4.1. Myopic Scenario

In the myopic scenario, both manufacturer and retailer act in a myopic way so that they only focus on the profit maximization in a finite foreseeable time interval [0,T]. In this scenario, the objective function of the manufacturer and retailer can be written as

(5) $$\begin{array}{cc} J_{M} & =\int_{0}^{T}\left(U_{M}D\left(t\right)-\frac{\mu_{M}}{2}E_{M}^{2}\left(t\right)-\phi \frac{\mu_{R}}{2}E_{R}^{2}\left(t\right)\right)dt, \end{array}$$

(6) $$\begin{array}{cc} J_{R} & =\int_{0}^{T}\left(U_{R}D\left(t\right)-\left(1-\phi \right)\frac{\mu_{R}}{2}E_{R}^{2}\left(t\right)\right)dt. \end{array}$$

Considering that this paper aims to study the advertising strategies with reference price, and without loss of generality, a given market price is assumed. Utilizing the principle of maximum and one-time decision-making process, we get the Hamiltonian equation as following

(7) $$\begin{array}{cc} H_{M} & =\max_{E_{M},E_{R}\geq 0}U_{M}\left(D_{0}+\alpha \left(r-p\right)+\beta W\right)-\frac{\mu_{M}}{2}E_{M}^{2}-\phi \frac{\mu_{R}}{2}E_{R}^{2}\\ \; & \;+\lambda_{1M}\left(\theta_{M}E_{M}+\theta_{R}E_{R}-\delta W\right)+\lambda_{2M}\left(\gamma (p-r)+\sigma W\right), \end{array}$$

(8) $$\begin{array}{cc} H_{R} & =\max_{E_{M},E_{R}\geq 0}U_{R}\left(D_{0}+\alpha \left(r-p\right)+\beta W\right)-\left(1-\phi \right)\frac{\mu_{R}}{2}E_{R}^{2}\\ \; & \;+\lambda_{1R}\left(\theta_{M}E_{M}+\theta_{R}E_{R}-\delta W\right)+\lambda_{2R}\left(\gamma (p-r)+\sigma W\right), \end{array}$$

where $\lambda_{1M}$ and $\lambda_{2M}$ are adjoint variables related to the goodwill and the reference price of the manufacturer respectively with $\lambda_{1M}\left(T\right)=\lambda_{2M}\left(T\right)=0$, the same meaning and setting as in these functions and they satisfy

(9) $$\begin{array}{cc} {\dot{\lambda }}_{1M}\left(t\right) & =-\frac{\partial H_{M}}{\partial W}=-\beta U_{M}+\delta \lambda_{1M}-\sigma \lambda_{2M}, \end{array}$$

(10) $$\begin{array}{cc} {\dot{\lambda }}_{2M}\left(t\right) & =-\frac{\partial H_{M}}{\partial r}=-\alpha U_{M}+\gamma \lambda_{1M}, \end{array}$$

(11) $$\begin{array}{cc} {\dot{\lambda }}_{1R}\left(t\right) & =-\frac{\partial H_{R}}{\partial W}=-\beta U_{R}+\delta \lambda_{1R}-\sigma \lambda_{2R}, \end{array}$$

(12) $$\begin{array}{cc} {\dot{\lambda }}_{2R}\left(t\right) & =-\frac{\partial H_{R}}{\partial r}=-\alpha U_{R}+\gamma \lambda_{2R}. \end{array}$$

Solve the differential equations above and we can get the optimal efforts on advertising of manufacturer and retailer, and the optimal cost subsidy which maximizes the Hamiltonian function is calculated as

(13) $$E_{M}^{*}=\frac{\lambda_{1M}\theta_{M}}{\mu_{M}},\;E_{R}^{*}=\frac{\lambda_{1R}\theta_{R}}{\left(1-\phi \right)\mu_{R}},\;\phi^{*}=\frac{2\lambda_{1M}-\lambda_{1R}}{2\lambda_{1M}+\lambda_{1R}},$$

where when γ≠δ, it can be calculated that

(14) $$\begin{array}{cc} \lambda_{1M} & =U_{M}\left(\frac{\beta \gamma +\alpha \sigma }{\delta \gamma }+e^{\gamma (t-T)}\frac{\alpha \sigma }{\gamma (\gamma -\delta )}-e^{\delta (t-T)}\left(\frac{\beta \gamma +\alpha \sigma }{\delta \gamma }+\frac{\alpha \sigma }{\gamma (\gamma -\delta )}\right)\right), \end{array}$$

(15) $$\begin{array}{ll} \lambda_{2M} & =U_{M}\frac{\alpha }{\gamma }\left(1-e^{\gamma (t-T)}\right), \end{array}$$

(16) $$\begin{array}{cc} \lambda_{1R} & =U_{R}\left(\frac{\beta \gamma +\alpha \sigma }{\delta \gamma }+e^{\gamma (t-T)}\frac{\alpha \sigma }{\gamma (\gamma -\delta )}-e^{\delta (t-T)}\left(\frac{\beta \gamma +\alpha \sigma }{\delta \gamma }+\frac{\alpha \sigma }{\gamma (\gamma -\delta )}\right)\right), \end{array}$$

(17) $$\begin{array}{ll} \lambda_{2R} & =U_{R}\frac{\alpha }{\gamma }\left(1-e^{\gamma (t-T)}\right). \end{array}$$

It is easy to verify that ${\dot{E}}_{M}\left(t\right)<0$, ${\dot{E}}_{R}\left(t\right)<0$, $E_{M}\left(T\right)=E_{R}\left(T\right)=0$, equally means that the equilibrium advertising efforts of all the participating members in supply chain are positive at first, and then decreasing as time goes by. In the end, both the manufacturer and the retailer will not advertise any more.

**Proposition** **1.** *Under the assumption of a given cost subsidy rate and γ≠δ, the optimal national advertising effort of the manufacturer and the optimal local advertising effort of the retailer in the situation of myopic Stackelberg game are*

(18) $$\begin{array}{cc} E_{M}^{*1}\left(t\right) & =U_{M}\frac{\theta_{M}}{\mu_{M}}\left(\frac{\beta \gamma +\alpha \sigma }{\delta \gamma }+e^{\gamma (t-T)}\frac{\alpha \sigma }{\gamma (\gamma -\delta )}-e^{\delta (t-T)}\left(\frac{\beta \gamma +\alpha \sigma }{\delta \gamma }+\frac{\alpha \sigma }{\gamma (\gamma -\delta )}\right)\right), \end{array}$$

(19) $$\begin{array}{cc} E_{R}^{*1}\left(t\right) & =U_{R}\frac{\theta_{R}}{\left(1-\phi \right)\mu_{R}}\left(\frac{\beta \gamma +\alpha \sigma }{\delta \gamma }+e^{\gamma (t-T)}\frac{\alpha \sigma }{\gamma (\gamma -\delta )}-e^{\delta (t-T)}\left(\frac{\beta \gamma +\alpha \sigma }{\delta \gamma }+\frac{\alpha \sigma }{\gamma (\gamma -\delta )}\right)\right). \end{array}$$

It can be proved that when γ=δ, for a given ϕ, with the calculation of the limitation theory, the optimal national and local advertising efforts share the same characteristics with Proposition 1.

**Corollary** **1.** *Under the assumption of a given price p, the equilibrium advertising efforts described in Proposition 1 satisfy*

- $E_{M}^{*1},E_{R}^{*1}$ decrease with time t, and when ασ<βδ is satisfied, $E_{M}^{*1},E_{R}^{*1}$ remain concave;
- For all the time and when γ>δ, there exists a positive relationship between $E_{M}^{*1},E_{R}^{*1}$ and parameters ασ,β, and a negative relationship between $E_{M}^{*1},E_{R}^{*1}$ and parameters μ,γ−δ;
- For all the time, there exists a positive relationship between $E_{R}^{*1}$ and the cost subsidy rate ϕ.

Proposition 1 and Corollary 1 indicate that the partner firms in supply chain should boost a high level of advertising efforts at first considering the effect of carryover and the results of the relevant sensitivity analysis. As time going by, both of the manufacturer and retailer should decrease their effort level to cut down the effort costs. It is because that the active advertising activities have the positive contribution to the goodwill and reference, which can lead to an increasing market demand. On the other hand, a concave effort cost can lead to a lower advertising effort level of participating members in supply chain. Considering the cost subsidy from manufacturer, retailer’s advertising effort can be enlarged, which shows that the manufacturer’s participation in local advertising activities has the positive incentive effect on the retailer.

**Proposition** **2.** *The optimal cost subsidy ϕ from the manufacturer to the retailer is*

(20) $$\phi^{*1}=\left\{\begin{array}{ll} \frac{2U_{M}-U_{R}}{2U_{M}+U_{R}}, & \;if\;\frac{U_{M}}{U_{R}}>\frac{1}{2},\\ 0, & \;otherwise. \end{array}\right.$$

Here the condition $U_{M}/U_{R}>1/2$ has the practical significance which means that as the critical role in the supply chain, manufacturer’s proportion of net profit should be higher than 1/3. On the contrary, if the manufacturer’s proportion is lower than that, the coordination process will not correspond to Pareto improvement rule, which will lead the manufacturer to have no motivation to improve coordination mechanism of the advertising in the supply chain. However, when the retailer is very capable of bargaining, which makes the manufacturer unprofitable, this phenomenon will occur in practice. Differentiating the equilibrium cost subsidy rate $\phi^{*1}$ from the manufacturer with respect to $U_{M},U_{R}$. We can get $\partial \phi^{*1}/\partial U_{M}>0$, $\partial \phi^{*1}/\partial U_{R}<0$ under the condition that $2U_{M}>U_{R}$. It implies that the higher (lower) the manufacturer’s (retailer’s) marginal profit is, the higher the cost subsidy rate will be, which is consistent to the practice.

Taking the equilibrium efforts into the corresponding differential equations mentioned above, we get the time path of accumulated goodwill and reference price on products as follows,

(21) $$\begin{array}{ll} W(t) & =W_{SS1}+\left(W_{0}-W_{SS1}\right)e^{-\delta t},\;W\left(0\right)=W_{0}. \end{array}$$

(22) $$\begin{array}{cc} r(t) & =r_{SS1}+\left(r_{0}-r_{SS1}\right)e^{-\gamma t}+\frac{\sigma \left(W_{0}-W_{SS1}\right)}{\gamma -\delta }\left(e^{-\delta t}-e^{-\gamma t}\right),\;r\left(0\right)=r_{0}. \end{array}$$

Here γ≠δ, and $W_{SS1},r_{SS1}$ are the steady states of the goodwill and the reference price respectively, given by

(23) $$W_{SS1}=\frac{\theta_{M}}{\delta }E_{M}^{*1}+\frac{\theta_{R}}{\delta }E_{R}^{*1},\;r_{SS1}=p+\frac{\sigma W_{SS1}}{\gamma }.$$

Then substitute the equilibriums above into the objective function of the manufacturer and the retailer in sequence, we can get their optimal profits as follows,

(24) $$\begin{array}{cc} J_{M}^{*} & =\int_{0}^{T}\left(U_{M}D^{*}-\frac{\mu_{M}}{2}{\left(E_{M}^{*}\right)}^{2}-\phi^{*}\frac{\mu_{R}}{2}{\left(E_{R}^{*}\right)}^{2}\right)dt, \end{array}$$

(25) $$\begin{array}{cc} J_{R}^{*} & =\int_{0}^{T}\left(U_{R}D^{*}-\left(1-\phi^{*}\right)\frac{\mu_{R}}{2}{\left(E_{R}^{*}\right)}^{2}\right)dt. \end{array}$$

### 4.2. Far-Sighted Scenario

In the far-sighted scenario, both manufacturer and retailer are far-sighted. The planning horizon of them can be extended to positive infinity. The objective function of the manufacturer and the retailer can be written as

(26) $$\begin{array}{cc} J_{M} & =\int_{0}^{\infty }e^{-\rho t}\left(U_{M}D\left(t\right)-\frac{\mu_{M}}{2}E_{M}^{2}\left(t\right)-\phi \frac{\mu_{R}}{2}E_{R}^{2}\left(t\right)\right)dt, \end{array}$$

(27) $$\begin{array}{cc} J_{R} & =\int_{0}^{\infty }e^{-\rho t}\left(U_{R}D\left(t\right)-\left(1-\phi \right)\frac{\mu_{R}}{2}E_{R}^{2}\left(t\right)\right)dt. \end{array}$$

Denote the equilibrium current profits of the manufacturer and the retailer after time t in Hamilton-Jacobi-Bellman Equation (HJB equation) are, then for all W≥0,r≥0, $V_{M},V_{R}$ should meet the equations below

(28) $$\begin{array}{cc} \rho V_{M} & =\max_{E_{M},E_{R}\geq 0}U_{M}\left(D_{0}+\alpha \left(r-p\right)+\beta W\right)-\frac{\mu_{M}}{2}E_{M}^{2}-\phi \frac{\mu_{R}}{2}E_{R}^{2}\\ \; & \;+V_{1M}^{{}^{\prime }}\left(\theta_{M}E_{M}+\theta_{R}E_{R}-\delta W\right)+V_{2M}^{{}^{\prime }}\left(\gamma (p-r)+\sigma W\right), \end{array}$$

(29) $$\begin{array}{cc} \rho V_{R} & =\max_{E_{M},E_{R}\geq 0}U_{R}\left(D_{0}+\alpha \left(r-p\right)+\beta W\right)-\left(1-\phi \right)\frac{\mu_{R}}{2}E_{R}^{2}\\ \; & \;+V_{1R}^{{}^{\prime }}\left(\theta_{M}E_{M}+\theta_{R}E_{R}-\delta W\right)+V_{2R}^{{}^{\prime }}\left(\gamma (p-r)+\sigma W\right). \end{array}$$

We differentiate HJB equation with respect to $E_{M},E_{R}$ respectively and the optimal efforts of manufacturer and retailer can be expressed as

(30) $$E_{M}^{*}=\frac{V_{1M}^{{}^{\prime }}\theta_{M}}{\mu_{M}},\;E_{R}^{*}=\frac{V_{1R}^{{}^{\prime }}\theta_{R}}{\left(1-\phi \right)\mu_{R}},\;\phi^{*}=\frac{2V_{1M}^{{}^{\prime }}-V_{1R}^{{}^{\prime }}}{2V_{1M}^{{}^{\prime }}+V_{1R}^{{}^{\prime }}},$$

where $V_{1M}^{{}^{\prime }},V_{2M}^{{}^{\prime }},V_{1R}^{{}^{\prime }},V_{2R}^{{}^{\prime }}$ can be calculated as

(31) $$\begin{array}{cc} V_{1M}^{{}^{\prime }} & =\frac{\beta U_{M}}{\rho +\delta }+\frac{\alpha \sigma U_{M}}{\left(\rho +\delta )(\rho +\gamma \right)},\;V_{2M}^{{}^{\prime }}=\frac{\alpha U_{M}}{\rho +\gamma }, \end{array}$$

(32) $$\begin{array}{cc} V_{1R}^{{}^{\prime }} & =\frac{\beta U_{R}}{\rho +\delta }+\frac{\alpha \sigma U_{R}}{\left(\rho +\delta )(\rho +\gamma \right)},\;V_{2R}^{{}^{\prime }}=\frac{\alpha U_{R}}{\rho +\gamma }. \end{array}$$

**Proposition** **3.** *Under the assumption of a given cost subsidy rate ϕ, the optimal national advertising effort of the manufacturer and the optimal local advertising effort of the retailer in the situation of far-sighted Stackelberg game are*

(33) $$\begin{array}{cc} E_{M}^{*2} & =\frac{\theta_{M}}{\mu_{M}}\left(\frac{\beta U_{M}}{\rho +\delta }+\frac{\alpha \sigma U_{M}}{\left(\rho +\delta )(\rho +\gamma \right)}\right), \end{array}$$

(34) $$\begin{array}{cc} E_{R}^{*2} & =\frac{\theta_{R}}{\left(1-\phi \right)\mu_{R}}\left(\frac{\beta U_{R}}{\rho +\delta }+\frac{\alpha \sigma U_{R}}{\left(\rho +\delta )(\rho +\gamma \right)}\right). \end{array}$$

**Proposition** **4.** *The manufacturer’s optimal cost subsidy ϕ to the retailer is*

(35) $$\phi^{*2}=\left\{\begin{array}{cc} \frac{2U_{M}-U_{R}}{2U_{M}+U_{R}}, & \;if\;\frac{U_{M}}{U_{R}}>\frac{1}{2},\\ 0, & \;otherwise. \end{array}\right.$$

Propositions 3 and 4 illustrate the following consequences:

- When ϕ=0, efforts of manufacturer and retailer include two parts. One part depicts the relationship between their efforts and market demand in the aspect of goodwill. The other part indicates the relationship between their efforts and market demand from the perspective of reference price. Especially, when σ=0, the second part will be equal to 0. It means that goodwill is the critical link between advertising efforts and reference price. However, in practice the consumer’s perception of reference price can be influenced by product’s goodwill. Then σ>0 is common. Under this situation, partner firms tend to make more efforts on collaborative advertising.
- As
  (36) $$\phi^{*2}=\frac{2U_{M}-U_{R}}{2U_{M}+U_{R}},\;\frac{U_{M}}{U_{R}}>\frac{1}{2},$$
  it can be indicated that
  (37) $$\frac{\partial E_{M}^{*2}}{\partial U_{M}}>0,\;\frac{\partial E_{R}^{*2}}{\partial U_{R}}>0,\;\frac{\partial E_{M}^{*2}}{\partial U_{R}}=0,\;\frac{\partial E_{R}^{*2}}{\partial U_{M}}>0.$$
  Equations about the manufacturer indicate that a higher marginal profit of manufacturer will lead to a higher level of advertising effort of manufacturer and retailer. It shares the same rule with the impact of retailer’s marginal profit on her advertising effort. However, retailer’s marginal profit has no relationship with manufacturer’s advertising effort.
- The positive relationship between manufacturer’s cost subsidy and retailer’s advertising effort is the same as the relevant rule in myopic scenario. However, it is limited by the condition that $U_{M}/U_{R}>1/2$.
- When the participating members in supply chain only focus on the immediate profit, which is represented as ρ=0, the equilibrium advertising efforts in the infinite situation is equivalent to that in the finite situation when T→∞. It shows that when the time interval approaches to the positive infinity, the equilibrium advertising efforts in both of the myopic scenario and the far-sighted scenario can be the same.

Taking the equilibrium efforts into the corresponding differential equations mentioned above, we get the time path of accumulated goodwill and reference price on products as follows,

(38) $$\begin{array}{cc} W(t) & =W_{SS2}+\left(W_{0}-W_{SS1}\right)e^{-\delta t},\;W\left(0\right)=W_{0}. \end{array}$$

(39) $$\begin{array}{cc} r(t) & =r_{SS2}+\left(r_{0}-r_{SS2}\right)e^{-\gamma t}+\frac{\sigma \left(W_{0}-W_{SS2}\right)}{\gamma -\delta }\left(e^{-\delta t}-e^{-\gamma t}\right),\;r\left(0\right)=r_{0}. \end{array}$$

Here γ≠δ, and $W_{SS2},r_{SS2}$ are the steady states of the goodwill and the reference price respectively, given by

(40) $$W_{SS2}=\frac{\theta_{M}}{\delta }E_{M}^{*2}+\frac{\theta_{R}}{\delta }E_{R}^{*2},\;r_{SS2}=p+\frac{\sigma W_{SS2}}{\gamma }.$$

Then substitute the equilibriums above into the objective function of the manufacturer and the retailer in sequence, we can get their optimal profits as follows,

(41) $$\begin{array}{cc} J_{M}^{*} & =\left(W_{0}-W_{SS2}\right)\left(\frac{\beta U_{M}}{\rho +\delta }+\frac{\alpha \sigma U_{M}}{\left(\rho +\delta )(\rho +\gamma \right)}\right)+\left(r_{0}-r_{SS2}\right)\left(\frac{\alpha U_{M}}{\rho +\gamma }\right)\\ \; & \;+\frac{U_{M}\left(D_{0}+\alpha \left(r_{SS2}-p\right)+\beta W_{SS2}\right)}{\rho }-\frac{\mu_{M}}{2\rho }{\left(E_{M}^{*2}\right)}^{2}-\phi \frac{\mu_{R}}{2\rho }{\left(E_{R}^{*2}\right)}^{2}, \end{array}$$

(42) $$\begin{array}{cc} J_{R}^{*} & =\left(W_{0}-W_{SS2}\right)\left(\frac{\beta U_{R}}{\rho +\delta }+\frac{\alpha \sigma U_{R}}{\left(\rho +\delta )(\rho +\gamma \right)}\right)+\left(r_{0}-r_{SS2}\right)\left(\frac{\alpha U_{R}}{\rho +\gamma }\right)\\ \; & \;+\frac{U_{R}\left(D_{0}+\alpha \left(r_{SS2}-p\right)+\beta W_{SS2}\right)}{\rho }-\left(1-\phi \right)\frac{\mu_{R}}{2\rho }{\left(E_{R}^{*2}\right)}^{2}. \end{array}$$

## 5. Cooperative Game Situations

In the cooperative game, both the manufacturer and the retailer can reach a vertical binding contract to cooperate with each other and make decisions in accordance with the profit maximization of the whole supply chain, which is the benchmark of the game equilibrium. Same as the analyses in Section 4, in this section we will also pay attention to the difference of equilibriums between the myopic scenario and the far-sighted scenario.

### 5.1. Myopic Scenario

In the myopic scenario, both manufacturer and retailer in supply chain make centralized decision, but they act in a myopic way so that they only focus on the profit maximization of the whole supply chain in a finite foreseeable time interval [0,T]. In this scenario, the objective function of the whole supply chain can be written as

(43) $$J_{S}=\int_{0}^{T}\left(\left(U_{M}+U_{R}\right)D\left(t\right)-\frac{\mu_{M}}{2}E_{M}^{2}\left(t\right)-\frac{\mu_{R}}{2}E_{R}^{2}\left(t\right)\right)dt.$$

Considering that this paper aims to study the advertising strategies with reference price, which is the same as situations in Section 4. Without loss of generality, a given market price is assumed here. Utilizing the principle of maximum and one-time decision-making process, we get the Hamiltonian equation as follows,

(44) $$\begin{array}{cc} H_{S} & =\max_{E_{M},E_{R}\geq 0}\left(U_{M}+U_{R}\right)\left(D_{0}+\alpha \left(r-p\right)+\beta W\right)-\frac{\mu_{M}}{2}E_{M}^{2}-\phi \frac{\mu_{R}}{2}E_{R}^{2}\\ \; & \;+\lambda_{1S}\left(\theta_{M}E_{M}+\theta_{R}E_{R}-\delta W\right)+\lambda_{2S}\left(\gamma (p-r)+\sigma W\right), \end{array}$$

where $\lambda_{1S}$ and $\lambda_{2S}$ are adjoint variables related to the goodwill and the reference price of the manufacturer respectively with $\lambda_{1S}\left(T\right)=\lambda_{2S}\left(T\right)=0$, and they satisfy

(45) $$\begin{array}{cc} {\dot{\lambda }}_{1S}\left(t\right) & =-\frac{\partial H_{S}}{\partial W}=-\beta \left(U_{M}+U_{R}\right)+\delta \lambda_{1S}-\sigma \lambda_{2S}, \end{array}$$

(46) $$\begin{array}{cc} {\dot{\lambda }}_{2S}\left(t\right) & =-\frac{\partial H_{S}}{\partial r}=-\alpha \left(U_{M}+U_{R}\right)+\gamma \lambda_{2S}. \end{array}$$

According to the maximum principle, the optimal advertising efforts of manufacturer and retailer which maximize the Hamiltonian function are calculated as

(47) $$E_{M}^{*}=\frac{\lambda_{1S}\theta_{M}}{\mu_{M}},\;E_{R}^{*}=\frac{\lambda_{1S}\theta_{R}}{\mu_{R}},$$

where when γ≠δ, it can be calculated that

(48) $$\begin{array}{cc} \lambda_{1S} & =\left(U_{M}+U_{R}\right)\left(\frac{\beta \gamma +\alpha \sigma }{\delta \gamma }+e^{\gamma (t-T)}\frac{\alpha \sigma }{\gamma (\gamma -\delta )}-e^{\delta (t-T)}\left(\frac{\beta \gamma +\alpha \sigma }{\delta \gamma }+\frac{\alpha \sigma }{\gamma (\gamma -\delta )}\right)\right), \end{array}$$

(49) $$\begin{array}{cc} \lambda_{2S} & =\left(U_{M}+U_{R}\right)\frac{\alpha }{\gamma }\left(1-e^{\gamma (t-T)}\right). \end{array}$$

It is easy to verify that ${\dot{E}}_{M}\left(t\right)<0$, ${\dot{E}}_{R}\left(t\right)<0$, $E_{M}\left(T\right)=E_{R}\left(T\right)=0$, equally means that the equilibrium advertising efforts of all the participating members in supply chain are positive at first, and then decreasing as time goes by. In the end, both the manufacturer and the retailer will not work on the advertising.

**Proposition** **5.** *Under the assumption of γ≠δ, the optimal national advertising effort of the manufacturer and the optimal local advertising effort of the retailer in the situation of myopic cooperative game are*

(50) $$\begin{array}{cc} E_{M}^{*3}\left(t\right) & =\left(U_{M}+U_{R}\right)\frac{\theta_{M}}{\mu_{M}}\left(\frac{\beta \gamma +\alpha \sigma }{\delta \gamma }+e^{\gamma (t-T)}\frac{\alpha \sigma }{\gamma (\gamma -\delta )}\right.\\ \; & \;\left.-e^{\delta (t-T)}\left(\frac{\beta \gamma +\alpha \sigma }{\delta \gamma }+\frac{\alpha \sigma }{\gamma (\gamma -\delta )}\right)\right), \end{array}$$

(51) $$\begin{array}{cc} E_{R}^{*3}\left(t\right) & =\left(U_{M}+U_{R}\right)\frac{\theta_{R}}{\mu_{R}}\left(\frac{\beta \gamma +\alpha \sigma }{\delta \gamma }+e^{\gamma (t-T)}\frac{\alpha \sigma }{\gamma (\gamma -\delta )}\right.\\ \; & \;\left.-e^{\delta (t-T)}\left(\frac{\beta \gamma +\alpha \sigma }{\delta \gamma }+\frac{\alpha \sigma }{\gamma (\gamma -\delta )}\right)\right). \end{array}$$

It can be proved that when γ=δ, with the calculation of the limitation theory, the optimal national and local advertising efforts share the same characteristics with Proposition 1.

Comparing Proposition 1 with 5, it is indicated that when manufacturer and retailer are myopic, manufacturer’s effort in the situation of cooperative game is higher than that in the situation of Stackelberg game (namely, $E_{M}^{*1}<E_{M}^{*3}$ ). Retailer’s effort depends on the relationship between between 1/(1−ϕ) and $U_{M}+U_{R}$. And

(52) $$E_{R}^{*1}-E_{R}^{*3}=-\frac{U_{R}\theta_{R}}{2\mu_{R}}\Delta <0,$$

can be obtained, where

(53) $$\Delta =\frac{\beta \gamma +\alpha \sigma }{\delta \gamma }+e^{\gamma (t-T)}\frac{\alpha \sigma }{\gamma (\gamma -\delta )}-e^{\delta (t-T)}\left(\frac{\beta \gamma +\alpha \sigma }{\delta \gamma }+\frac{\alpha \sigma }{\gamma (\gamma -\delta )}\right)>0.$$

Hence Corollary 2 can be given as following.

**Corollary** **2.** *The one time decision-making efforts on advertising of manufacturer and retailer in cooperative game situation are higher than that in the situation of Stackelberg game, i.e.,*

(54) $$E_{M}^{*1}<E_{M}^{*3},\;E_{R}^{*1}<E_{R}^{*3}.$$

Taking the equilibrium efforts into the corresponding differential equations mentioned above, we get the time path of accumulated goodwill and reference price on products as follows,

(55) $$\begin{array}{cc} W(t) & =W_{SS3}+\left(W_{0}-W_{SS3}\right)e^{-\delta t},\;W\left(0\right)=W_{0}. \end{array}$$

(56) $$\begin{array}{cc} r(t) & =r_{SS3}+\left(r_{0}-r_{SS3}\right)e^{-\gamma t}+\frac{\sigma \left(W_{0}-W_{SS3}\right)}{\gamma -\delta }\left(e^{-\delta t}-e^{-\gamma t}\right),\;r\left(0\right)=r_{0}. \end{array}$$

Here γ≠δ, and $W_{SS3},r_{SS3}$ are the steady states of the goodwill and the reference price respectively, given by

(57) $$W_{SS3}=\frac{\theta_{M}}{\delta }E_{M}^{*3}+\frac{\theta_{R}}{\delta }E_{R}^{*3},\;r_{SS3}=p+\frac{\sigma W_{SS3}}{\gamma }.$$

Comparing the results above with that in the myopic situation of Stackelberg game correspondingly, it can be found that when both the manufacturer and the retailer act as one centralized role, the equilibrium goodwill and reference price will increase, because of the higher optimal national and local advertising efforts. Then substitute the equilibriums above into the objective function of the manufacturer and the retailer in sequence, we can get their optimal profits as follows,

(58) $$\begin{array}{cc} J_{S}^{*} & =\int_{0}^{T}\left(\left(U_{M}+U_{R}\right)D^{*}-\frac{\mu_{M}}{2}{\left(E_{M}^{*}\right)}^{2}-\frac{\mu_{R}}{2}{\left(E_{R}^{*}\right)}^{2}\right)dt. \end{array}$$

### 5.2. Far-Sighted Scenario

In the far-sighted scenario, we further extend the planning horizon of the manufacturer and retailer to positive infinity and assume that both of them are far-sighted to explore the long-term behaviors of the whole supply chain. The objective function of the manufacturer and the retailer can be written as

(59) $$\begin{array}{cc} J_{S} & =\int_{0}^{\infty }e^{-\rho t}\left(\left(U_{M}+U_{R}\right)D\left(t\right)-\frac{\mu_{M}}{2}E_{M}^{2}\left(t\right)-\frac{\mu_{R}}{2}E_{R}^{2}\left(t\right)\right)dt. \end{array}$$

Denote the optimal current profit of the whole supply chain after time *t* in HJB equation is $V_{S}$, then for all W≥0,r≥0, $V_{S}$ should meet the equations below,

(60) $$\begin{array}{cc} \rho V_{S} & =\max_{E_{M},E_{R}\geq 0}\left(U_{M}+U_{R}\right)\left(D_{0}+\alpha \left(r-p\right)+\beta W\right)-\frac{\mu_{M}}{2}E_{M}^{2}-\frac{\mu_{R}}{2}E_{R}^{2}\\ \; & \;+V_{1S}^{{}^{\prime }}\left(\theta_{M}E_{M}+\theta_{R}E_{R}-\delta W\right)+V_{2S}^{{}^{\prime }}\left(\gamma (p-r)+\sigma W\right). \end{array}$$

We differentiate HJB equation with respect to $E_{M},E_{R}$ respectively and the optimal efforts of manufacturer and retailer can be expressed as

(61) $$E_{M}^{*}=\frac{V_{1S}^{{}^{\prime }}\theta_{M}}{\mu_{M}},\;E_{R}^{*}=\frac{V_{1S}^{{}^{\prime }}\theta_{R}}{\mu_{R}},$$

where $V_{1S}^{{}^{\prime }},V_{2S}^{{}^{\prime }}$ can be calculated as

(62) $$\begin{array}{cc} V_{1S}^{{}^{\prime }} & =\frac{\beta (U_{M}+U_{R})}{\rho +\delta }+\frac{\alpha \sigma (U_{M}+U_{R})}{\left(\rho +\delta )(\rho +\gamma \right)},\;V_{2S}^{{}^{\prime }}=\frac{\alpha (U_{M}+U_{R})}{\rho +\gamma }. \end{array}$$

**Proposition** **6.** *In the situation of far-sighted cooperative game, the optimal advertising efforts of the manufacturer and the retailer are*

(63) $$\begin{array}{cc} E_{M}^{*4} & =\frac{V_{1S}^{{}^{\prime }}\theta_{M}}{\mu_{M}}=\frac{\theta_{M}}{\mu_{M}}\left(\frac{\beta (U_{M}+U_{R})}{\rho +\delta }+\frac{\alpha \sigma (U_{M}+U_{R})}{\left(\rho +\delta )(\rho +\gamma \right)}\right), \end{array}$$

(64) $$\begin{array}{cc} E_{R}^{*4} & =\frac{V_{1S}^{{}^{\prime }}\theta_{R}}{\mu_{R}}=\frac{\theta_{R}}{\mu_{R}}\left(\frac{\beta (U_{M}+U_{R})}{\rho +\delta }+\frac{\alpha \sigma (U_{M}+U_{R})}{\left(\rho +\delta )(\rho +\gamma \right)}\right). \end{array}$$

Compare Proposition 3 with Proposition 6, it can be found that when manufacturer and retailer are far-sighted, manufacturer’s effort in the situation of cooperative game is higher than that in the situation of Stackelberg game (namely, $E_{M}^{*2}<E_{M}^{*4}$). Retailer’s effort depends on the relationship between 1/(1−ϕ) and $U_{M}+U_{R}$. And

(65) $$E_{R}^{*2}-E_{R}^{*4}=-\frac{\left(\alpha \sigma +\beta \gamma +\beta \rho \right)\theta_{R}U_{R}}{2\left(\rho +\delta \right)\left(\rho +\gamma \right)\mu_{R}}<0,$$

can be obtained. Hence Corollary 3 can be given as following.

**Corollary** **3.** *The long-term efforts on advertising of manufacturer and retailer in cooperative game situation are higher than those in the situation of Stackelberg game, i.e.,*

(66) $$E_{M}^{*2}<E_{M}^{*4},\;E_{R}^{*2}<E_{R}^{*4}.$$

Taking the equilibrium efforts into the corresponding differential equations mentioned above, we get the time path of accumulated goodwill and reference price on products as follows,

(67) $$\begin{array}{cc} W(t) & =W_{SS4}+\left(W_{0}-W_{SS4}\right)e^{-\delta t},\;W\left(0\right)=W_{0}. \end{array}$$

(68) $$\begin{array}{cc} r(t) & =r_{SS4}+\left(r_{0}-r_{SS4}\right)e^{-\gamma t}+\frac{\sigma \left(W_{0}-W_{SS4}\right)}{\gamma -\delta }\left(e^{-\delta t}-e^{-\gamma t}\right),\;r\left(0\right)=r_{0}. \end{array}$$

Here γ≠δ, and $W_{SS4},r_{SS4}$ are the steady states of the goodwill and the reference price respectively, given by

(69) $$W_{SS4}=\frac{\theta_{M}}{\delta }E_{M}^{*4}+\frac{\theta_{R}}{\delta }E_{R}^{*4},\;r_{SS4}=p+\frac{\sigma W_{SS4}}{\gamma }.$$

Comparing the results above with that in the far-sighted situation of Stackelberg game correspondingly, it can be found that when the manufacturer and the retailer act as one centralized role, the equilibrium goodwill and reference price will increase, because of the higher optimal national and local advertising efforts.

Then substitute the equilibria above into the objective function of the manufacturer and the retailer in sequence, we can get their optimal profits as follows,

(70) $$\begin{array}{cc} J_{S}^{*} & =\left(W_{0}-W_{SS4}\right)\left(\frac{\beta \left(U_{M}+U_{R}\right)}{\rho +\delta }+\frac{\alpha \sigma \left(U_{M}+U_{R}\right)}{\left(\rho +\delta )(\rho +\gamma \right)}\right)+\left(r_{0}-r_{SS4}\right)\left(\frac{\alpha \left(U_{M}+U_{R}\right)}{\rho +\gamma }\right)\\ \; & \;+\frac{\left(U_{M}+U_{R}\right)\left(D_{0}+\alpha \left(r_{SS4}-p\right)+\beta W_{SS4}\right)}{\rho }-\frac{\mu_{M}}{2\rho }{\left(E_{M}^{*4}\right)}^{2}-\frac{\mu_{R}}{2\rho }{\left(E_{R}^{*4}\right)}^{2}. \end{array}$$

### 5.3. Coordination Contract

Considering that the equilibrium in cooperative game situation is a benchmark in theory and there exists a higher total profit in the cooperative game situation, we need to conduct coordination to reach the optimal strategies in practice. However, as the cooperative game situation is just a kind of system in theory, it is hard to be conducted in practice without any coordination mechanisms. Therefore, to let the participating members in supply chain make decisions consistent to the optimal ones, a coordination contract between them should be designed and conducted. Here we only put forward the framework of coordination in the aspect of the revenue-sharing mechanism. In the studying category of cooperative game, the highlight is to construct the solution to cooperative game, i.e., the revenue/cost-sharing problems of the cooperative alliance. The common solutions to the game include Shapley value, core, bargaining set and so on. Considering the characteristics of our game model and the status gap between the manufacturer and the retailer, we introduce the Nash bargaining model to allocate the cooperative revenue.

According to the Nash bargaining model, in the situation of cooperative game, the allocated revenue should be the multiplication of both utility functions which maximize the revenue increments. Here we take far-sighted situation as an example to show the revenue sharing contract. The total revenue increment follows $\Delta J=J_{S}-J_{M}-J_{R}$. The manufacturer and the retailer negotiate with each other and allocate the cooperative revenue in the purpose of “win-win”. As we assume both of them are risk averter, the utility functions of them can be expressed as

(71) $$F_{M}\left(\Delta J_{M}\right)=1-e^{-\Psi_{M}\Delta J_{M}},\;F_{R}\left(\Delta J_{R}\right)=1-e^{-\Psi_{R}\Delta J_{R}},$$

where $\Psi_{M},\Psi_{R}$ indicate the proportion of revenue allocated to the manufacturer and the retailer respectively. Based on the Nash bargaining model, the optimal allocated revenues are the solution to the following equation,

(72) $$\begin{array}{cc} \; & \max_{\Delta J_{M},\Delta J_{R}}\;F\left(\Delta J_{M},\Delta J_{R}\right)=F_{M}\left(\Delta J_{M}\right)F_{R}\left(\Delta J_{R}\right)\\ \; & \;s.t.\;\Delta J_{M}+\Delta J_{R}=\Delta J. \end{array}$$

We can solve the optimal proportion of revenue allocation by the principle of first-order partial derivative, i.e., $\partial F/\partial \Delta J_{M}=0,\partial F/\partial \Delta J_{R}=0$. Taking the limitation of space and the focus of our paper into consideration, the specific solution will not be presented here. Therefore, this revenue-sharing contract can motivate the initiate of cooperation in the whole supply chain.

## 6. Numerical Analysis

Based on the theoretical analysis above, in this section, the numerical analyses will be conducted to further explore the rules in the optimal strategies. The data were collected from a real company (F Group) in Jiangsu Province, China. The main business of F Group is to produce and sell the steel cables. According to the practical survey of F Group and its retailer, their marginal profits follow that $U_{M}=4,U_{R}=3$, and other parameters can be assigned as $W_{0}=10$, $r_{0}=15$, $D_{0}=20$, T=1, α=5, β=3, δ=0.5, γ=0.3, σ=0.2, ρ=0.1, $\theta_{M}=0.5$, $\theta_{R}=0.3$, $\mu_{M}=8$, $\mu_{R}=6$.

**Figure 2:**
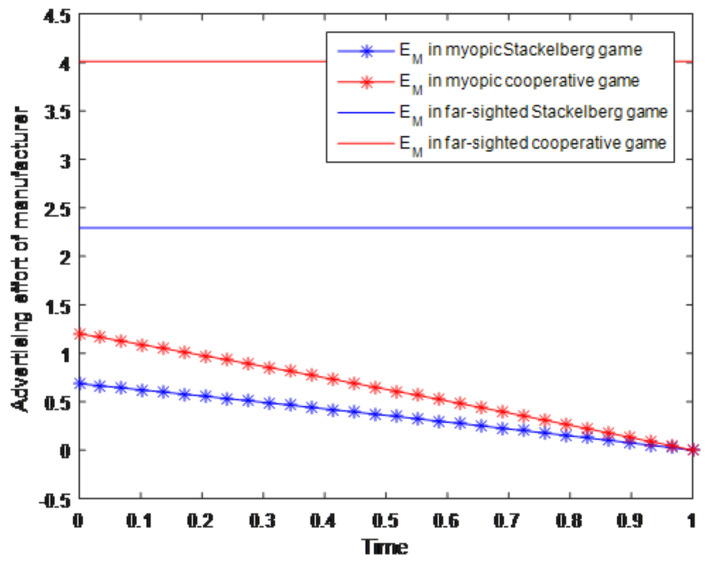
Advertising effort of manufacturer.

**Figure 3:**
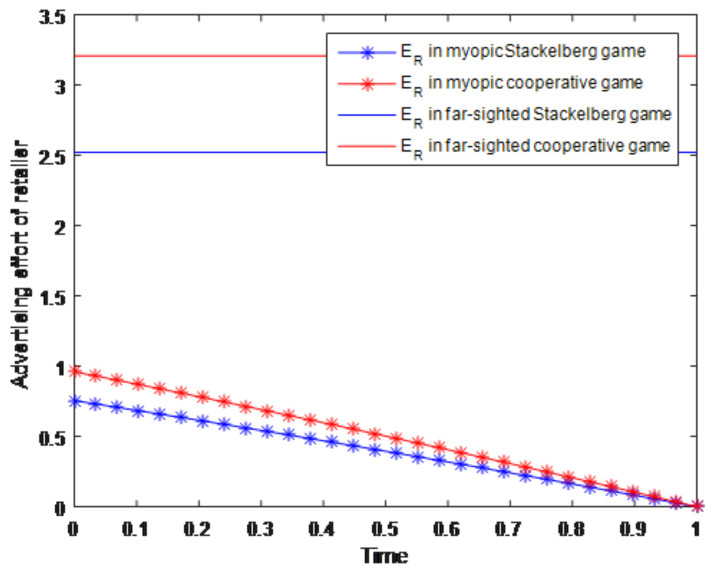
Advertising effort of retailer.

Two different groups of simulation experiments about the advertising effort of manufacturer and the retailer considering the myopic/far-sighted behaviors are shown in Figure 2 and Figure 3. In these two figures, the blue lines with asterisks represent the advertising effort of the manufacturer/retailer in the myopic Stackelberg game situation, while the red lines with asterisks represent the advertising effort of the manufacturer/retailer in the myopic cooperative game situation. Then the blue solid lines represent the advertising effort of the manufacturer/retailer in the far-sighted Stackelberg game situation, and the red solid lines represent the advertising effort of the manufacturer/retailer in the far-sighted cooperative game situation. Based on the simulations about the advertising effort of manufacturer in Figure 2, we can know that in the far-sighted situation, including both the Stackelberg game and cooperative game, the optimal effort of manufacturer remains stable over time. It indicates that if the manufacturer is far-sighted, which means that he will focus on the long-term profit maximization, he will make a high level effort of advertising initially and maintain this level to the end. However, when the manufacturer is myopic, which means that he will only focus on the profit maximization in an immediate time interval, he will decrease his effort level as time goes on in this short interval. In this way, the effort level in myopic situation varies with time and generally is decreasing. On the other hand, from Figure 1 we can also know that the effort level of the manufacturer in cooperative game situation (whether it is far-sighted or myopic) is higher than that in the Stackelberg game situation. In the far-sighted situation, this kind of increment is reflected in the intercept; while in the myopic situation, it is reflected in the slope. Likewise, the laws of far-sighted and myopic efforts of retailer in Stackelberg game and cooperative game in Figure 3 are the same as the manufacturer efforts in Figure 2.

The sensitivity analysis of several critical parameters including $\alpha ,\beta ,\mu_{M},\delta ,\gamma ,\sigma$ are conducted in this section and the results are shown in Table 2 as follow. It indicates that the optimal profits increase with α,β,σ, but decrease with $\mu_{M},\delta ,\gamma$ in both of the myopic and far-sighted situations. And the discount rate ρ in far-sighted situations has a negative relationship with the profits. Both of the parameters α,β show the advertising promotion effect on the market demand, which represents the gap between reference price and market price, and the goodwill respectively. A positive coefficient of parameter shows that the higher the goodwill is, the higher the reference price will be. Then the difference in reference price and market price will become larger, which leads to a higher demand and increasing profits. On the other hand, parameters $\mu_{M},\delta ,\gamma$ share the negative relationships with profits, which mean that the higher the parameters $\mu_{M},\delta ,\gamma$ are, the lower the profit is. Both of the parameters $\mu_{M},\mu_{R}$ represent the cost of effort for participating members, so we omit the sensitivity analysis of $\mu_{R}$ in the myopic situation. Since $\mu_{M}$ captures the effort cost, a higher cost will lead to a lower effort, so the profit will decrease correspondingly. Parameters δ,γ represent the decay rate of goodwill and reference price respectively. When there is no advertising effort on goodwill promotion (advertising), the goodwill of participating members will decay for the reason that the consumers will forget it as time going by, which is similar to the mechanism of reference price. The negative relationship indicates that a higher decay rate will lead to a lower profit and it is meaningful in practice, since companies should maintain a stable level of brand propagation and exposure to promote sales. Finally, a negative relationship between ρ and the optimal profit in the far-sighted situation indicates that for the patient consumers, considering that they usually pay more attention to the advertising, the manufacturer and the retailer should work harder on the advertising to increase the profit than that when the consumers are not patient.

## 7. Conclusions

Faced with dynamic business environment, it would be beneficial for the firms in the supply chain to act as far-sighted decision makers in theory. However, in practice, many firms tend to make the myopic decisions to maximize their short-term profit. Such behavior preferences of the partner firms in the supply chain have significant impacts on their optimal strategies including collaborative advertising, dynamic pricing and so on. This paper introduces the concept of time and uses the differential game to study the myopic/far-sighted behaviors in collaborative advertising with reference price effect in the supply chain. Fully considering the impact of different game structures, the optimal efforts on national advertising of the manufacturer and the optimal efforts on local advertising of the retailer are studied in the situation of Stackelberg game and cooperative game respectively. The main conclusions of this paper include:

- Both the manufacturer’s national advertising effort and the retailer’s local advertising effort have significant influences on the product goodwill and they further affect consumers selection. To increase the total profit of supply chain, manufacturer and retailer should make more efforts on advertising;
- When the partner firms are myopic, they only care for the immediate interest in a short period. Under this situation, optimal advertising efforts of both manufacturer and retailer reduce with the time to 0 in the end of the time interval. While when they are far-sighted, the optimal efforts remain stable and present as a constant. So in the far-sighted situation, the profit of supply chain is higher;
- Cooperative game is better than Stackelberg game for manufacturer, retailer and supply chain. With a profit allocation contract based on Nash bargaining model, higher effort can be realized in the situation of cooperative game.

Although this study has contributed to the behavior analysis in the supply chain management literature, there are still some directions remaining for future research. For example, the retail and wholesale prices are not considered in this study. However, it will be more interesting and practical to analyze the pricing strategies. On the other hand, a supply chain consisting of one manufacturer and some competing retailers may be more meaningful.

## Figures and Tables

**Table 1:** Notations and definitions.

Notation	Definition
Decision variables of manufacturer and retailer	
$E_{M}\left(t\right)$	National advertising effort of manufacturer
$E_{R}\left(t\right)$	Local advertising effort of retailer
ϕ(t)	Cost subsidy rate from manufacturer to retailer on the local advertising
	effort, 0<ϕ(t)<1
Parameters and other variables	
$U_{M},U_{R}$	Marginal profit of manufacturer and retailer
$\mu_{M},\mu_{R}$	Cost parameter associated with the advertising efforts of manufacturer
	and retailer
$\theta_{M},\theta_{R}$	Coefficient associated with the effort on goodwill of the product in the
	function of goodwill, $\theta_{M}\geq 0,\theta_{R}\geq$
γ,σ	Coefficient associated with the difference between market price and
	reference price, and the goodwill in the function of reference price,
	γ≥0,σ≥0
α,β	Coefficient associated with the difference between reference price and
	market price, and the goodwill in the demand function, α≥0,β≥0
δ	Decay rate of the goodwill, δ≥0
ρ	Discount rate
*D*	Demand for the product at time *t*, with initial demand $D_{0}\geq 0$
p(t)	Market price at time *t*, with $p\left(0\right)=p_{0}>0$
W(t),r(t)	Goodwill and reference price at time *t*, with $W\left(0\right)=W_{0}\geq 0$,
	$r\left(0\right)=r_{0}\geq 0$
$J_{M},J_{R},J_{S}$	Objective function (which is expressed as net profit) of the manufacturer,
	retailer and the whole supply chain for t∈[0,+∞).

**Table 2:** Sensitivity analysis.

Coefficient	Value	Profits
Myopic Stackelberg game $\left(J_{M}^{*},J_{R}^{*}\right)$		
α	(5;7)	(241.88;181.78), (268.76;201.98)
β	(3;6)	(241.88;181.78), (337.00;254.08)
$\mu_{M}$	(8;15)	(241.88;181.78), (241.86;181.54)
δ	(0.5;1)	(241.88;181.78), (221.17;166.15)
γ	(0.3;0.6)	(241.88;181.78), (233.75;175.68)
σ	(0.2;0.5)	(241.88;181.78), (264.92;199.19)
Myopic cooperative game $J_{S}^{*}$		
α	(5;7)	(423.66;470.74)
β	(3;6)	(423.66;591.08)
$\mu_{M}$	(8;15)	(423.66;423.41)
δ	(0.5;1)	(423.66;387.32)
γ	(0.3;0.6)	(423.66;409.43)
σ	(0.2;0.5)	(423.66;464.12)
Far-sighted Stackelberg game $\left(J_{M}^{*},J_{R}^{*}\right)$		
α	(5;7)	+
β	(3;6)	+
$\mu_{M}$	(8;15)	−
$\mu_{R}$	(6;12)	−
δ	(0.5;1)	−
γ	(0.3;0.6)	−
σ	(0.2;0.5)	+
ρ	(0.1;0.3)	−
Far-signted cooperative game $J_{S}^{*}$		
α	(5;7)	+
β	(3;6)	+
$\mu_{M}$	(8;15)	−
$\mu_{R}$	(6;12)	−
δ	(0.5;1)	−
γ	(0.3;0.6)	−
σ	(0.2;0.5)	+
ρ	(0.1;0.3)	−

## Data Availability

Not applicable.
